# Lung-Seeking Metastases

**DOI:** 10.3390/cancers11071010

**Published:** 2019-07-19

**Authors:** Giulia M. Stella, Stefan Kolling, Silvia Benvenuti, Chandra Bortolotto

**Affiliations:** 1 Department of Medical Sciences and Infectious Diseases, Unit of Respiratory System Diseases, IRCCS Fondazione Policlinico San Matteo, 27100 Pavia, Italy; 2 Medical School, University of Pavia, 27100 Pavia, Italy; 3 Department of Molecular Therapeutics and Exploratory Research, Candiolo Cancer Institute, FPO-IRCCS, 10060 Candiolo (TO), Italy; 4 Department of Intensive Medicine, Unit of Radiology, IRCCS Fondazione Policlinico San Matteo, 27100 Pavia, Italy

**Keywords:** metastases, lung, metastatic niche, biomarkers, biomechanics

## Abstract

Metastases from different cancer types most often affect the lung parenchyma. Moreover, the lungs are among the most frequent sites of growth of metastatic masses of uncertain/unknown lineage of origin. Thus, with regards to pulmonary neoplastic parenchymal nodules, the critical issue is to determine if they are *IN* the lung or *OF* the lung. In this review, we highlight the clinical, instrumental and molecular features which characterize lung metastases, mainly focusing on recently advancing and emerging concepts regarding the metastatic niche, inflammation, angiogenesis, immune modulation and gene expression. A novel issue is related to the analysis of biomechanical forces which cooperate in the expansion of tumor masses in the lungs. We here aim to analyze the biological, genetic and pathological features of metastatic lesions to the lungs, here referred to as site of metastatic growth. This point should be a crucial part of the algorithm for a proper diagnostic and therapeutic approach in the era of personalized medicine.

## 1. Introduction

Metastatic dissemination is a feature of highly malignant progression which is responsible for poor prognosis of affected patients and eventually leads to their death. The molecular and cellular mechanisms underlying the different proclivities of metastatic spreading are the topic of constant debate and intense research efforts because they have important implications for our ability to predict, identify and eradicate life-threatening advanced disease. Most often, cancer diagnosis is made when multiple lesions have already spread from the primary tumor site. Growing evidence suggests that cancer cells can remotely prepare distant sites for subsequent colonization, by modulating organ-seeking vesicles [1] and pro-inflammatory cytokines [2]. On the other hand, the application of phylogenetic analysis to cancer metastatic subclones could be helpful in dissecting evolutionary history of cancer lineages [3]. The latter could ultimately lead to the identification of driver genetic lesions that will impact on clinical management of advanced disease. 

The lungs are the second most frequent site of metastatic growth from extra-thoracic malignancies, with pulmonary secondary lesions being detected in 20–54% of cases [4]. They result from both lymphatic and hematogenous spread. In the adult population, the most common primary tumors to result in pulmonary metastases include breast, colorectal and renal carcinoma, uterine leiomyosarcoma and head and neck carcinoma, whereas endobronchial lesions most often derive from colorectal, renal and lung cancer as well as being related to lymphomas. In some cases, the origin remains undetermined or cannot be identified at all. The latter should be classified as cancer of unknown primary (CUP). 

Here, we discuss and analyze the clinical, radiological and specific molecular features of secondary pulmonary masses as well as point out the biological and biomechanical properties of the lungs as sites of metastatic seeding. 

## 2. Clinical and Radiological Presentation of Pulmonary Metastases

**Figure 1:**
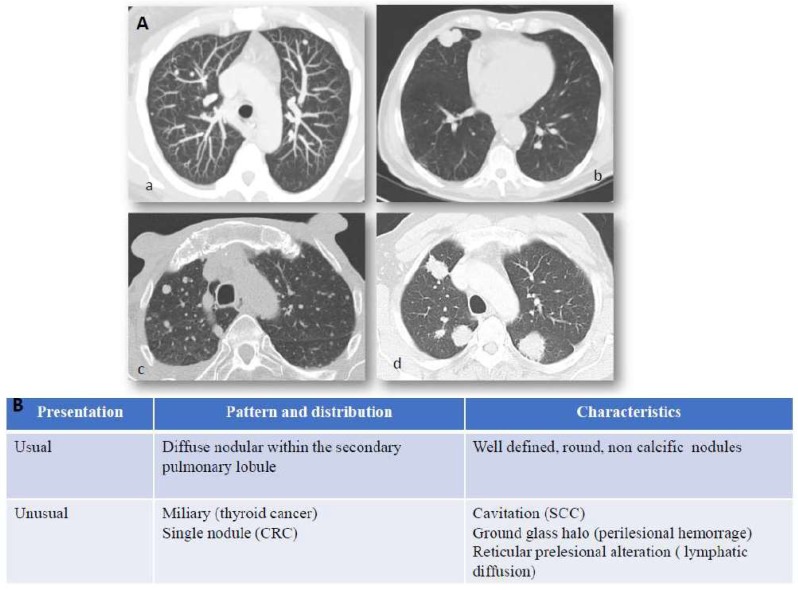
Lung metastases: imaging and immunostaining. In patients with known primary tumors, the appearance of multiple bilateral pulmonary nodules is highly suggestive of metastatic dissemination and no further invasive procedures are generally required to confirm diagnosis. Chest X-Ray (CXR) has shown little sensitivity over the years, relegating it to marginal use in low-risk patients or to be abandoned entirely as a surveillance method. While lung metastases can still be detected by CXR in specific scenarios, such as incidental findings in the emergency room, computed tomography is the main imaging modality employed for detection and follow-up of lung metastases. Panel (**A**) CT scan presentation of lung metastases: (**a**) usual features of lung metastases from colorectal cancer, (**b**) squamous cell lung cancer of unknown primary (CUP), (**c**) miliary distribution of secondary lesion from ovarian cancer, (**d**) multiple metastatic nodules from breast cancer. Hematogenous spread to the lungs most often characterizes those tumors which arise in organs with anatomical venous drainage towards the lungs, such as head and neck, thyroid, adrenals, kidneys, testes, melanoma, and osteosarcoma. Panel (**B**) Computed tomography (CT) presentation of lung metastases. Computed tomography is the main imaging modality employed for detection and follow-up of lung metastases. They generally appear as round and non-calcific nodules of variable dimensions, with smooth margins and a variable degree of vascularization. However, more unusual patterns can be detected as well (such as a miliary distribution or metastatization in the form of a single lung nodule). CRC = colorectal cancer, SCC = squamous cell carcinoma, CUP = Cancer of unknown primary.

Lung metastatic disease might display heterogeneous clinical characteristics and can occur with or without signs or symptoms. Similar to primary lung cancer, clinical manifestations of lung metastases are related to local growth of neoplastic masses and paraneoplastic syndromes. The characterization and follow-up of lung nodules in patients with known malignant disorders represent daily items in radiological practice. The imaging of pulmonary metastases is generally centered on surveillance with computed tomography (CT) (Figure 1, panel A). [5]. Lung metastatization is most often related to an endovascular spread of tumor cells nesting in the distal arterial pulmonary circulation. Due to this pattern of spread, lung metastases tend to be more abundant in basal and peripheral regions. Physiologically, as expressed by the ventilation/perfusion index, the basal regions are more highly vascularized, whereas the upper regions are more highly ventilated. Ultimately, this leads to hematogenous metastases being more numerous in the basal region. Details are described in Figure 1, panel B. The detection of a single pulmonary nodule in a patient with known malignancy may occur and a biopsy is required to differentiate between primary lung cancer and a solitary metastasis. Low-dose CT (LDCT) has constantly increased its performance in terms of image quality and dose reduction. Its use in substitution of chest-X-ray has been advocated since the early 2000s [6] but with mixed reports [7]. A more widespread use was made possible by the introduction of iterative reconstruction algorithms which became commercially available in 2009, and during the following decade have improved in availability and efficiency. Iterative reconstruction imaging protocol boosts the decrease in imaging noise at lower kV allowing a strong dose reduction with better image quality. An even stronger benefit is expected from the development of artificial intelligence reconstructed images [8]. The use of LDCT for lung tumor screening is already codified in the National Comprehensive Cancer Network NCCN guidelines [9] and is expected to be included in the future into guidelines to replace chest X-rays (CHR) in low-risk oncologic patients. For high-risk patients—receiving regular full-dose CT—the benefit of LDCT remains unclear but a more accurate evaluation over time without a significant dose increase may lead to inclusion into guidelines in the future. 

Magnetic resonance imaging (MRI) is becoming more and more sensitive in the diagnosis and follow-up of pulmonary metastases thanks to faster and more robust sequences for pulmonary use even with free-breathing approaches [10]. Ultrasound (US) plays no diagnostic role in the lung and can only support interventional transthoracic procedures—e.g., for lesions reaching the pleural surface- or for an endobronchial approach (EBUS). Imaging is not only used for diagnosis and follow-up of lung metastases but can also support and guide local treatment such as thermal treatments (generally CT-guided), namely radiofrequency, microwave, cryotherapy [11]. Nuclear medicine, especially with radiologic imaging fusion—positron emission tomography-computed tomography (PET-CT) and positron emission tomography-magnetic resonance imaging (PET-MRI), allows the coupling of morphologic data from imaging with physiological data on the uptake of radiotracers [12].

### Determining the Origin

**Figure 2:**
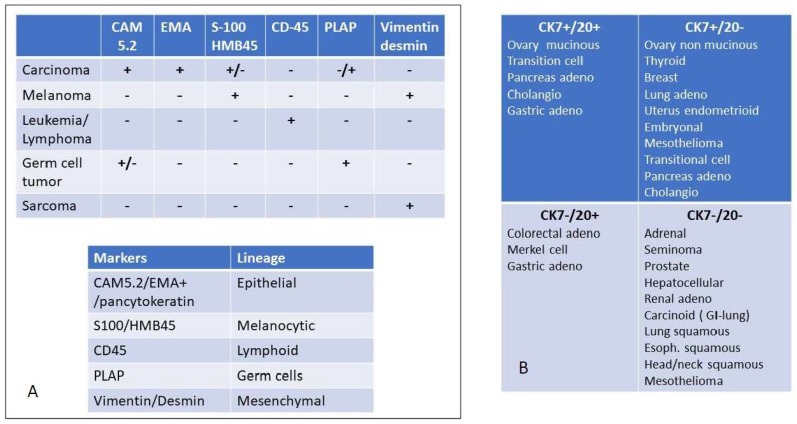
Immunohistochemical evaluation to detect the major origin of metastatic lesions: Panel (**A**) key screening antibody panel to detect the lineage of origin. It should be noted that melanoma is positive for vimentin but not for desmin, except for rare cases such as osteogenic melanoma which might express desmin [22] and sinovial mucosal melanoma [23]. In sarcoma desmin is not always expressed. For instance, solitary fibrous tumors and hemangioperycitoma like tumors are generally negative for desmin, as well as epitheliod sarcomas [24]. EMA = Epithelial Membrane Antigen, PLAP = Placenta Alkaline Phosphatase, Panel (**B**) once the diagnosis of carcinoma is reached, the cytokeratin expression may be useful to further delineate the tissue or organ of origin. The differential expression of CK 7 and CK 20 is among the most relevant discriminants of carcinomas of epithelial origin.

In case of detection of well differentiated malignant cells, their resemblance to the conventional morphology of non-cancerous cells can be helpful in identifying the lineage of origin; more commonly however, the metastases consist of moderately or poorly differentiated cells. Thus, immunohistochemistry (IHC) plays a crucial role in suggesting the site of origin. If the origin remains still evasive, an exhaustive diagnostic work-up should be initiated to determine the putative primary origin *versus* a metastatic dissemination from an occult site. In the case of lung lesions from an unknown primary site of origin, the pathologic approach should be *ad excludendum* to possibly identify the primary cancer of early metastatic dissemination. Nevertheless, tumor sequencing and gene signature profiling to determine the putative tissue of origin is still not recommended for routine clinical workup [9]. Morphology and IHC represent the more significant tools for the pathologist. In the era of molecular medicine, immunohistochemistry plays a relevant role in classifying tumor lineage (Figure 2): a screening IHC panel to analyze the major lineage of origin (epithelial, mesenchymal, lymphoid and melanocytic) most often provides major information to define the nature of an undifferentiated tumor. Many procedural algorithms related to IHC staining are available in the literature regarding both the identification of primary lung cancer [13] and the putative origin of secondary pulmonary lesions [14]. In case of epithelial-derived lesions, the expression of thyroid transcription factor 1 (TTF-1) is a highly specific marker for primary lung adenocarcinomas (ADCs) and it must be included in a panel of antibodies for the differential diagnosis between primary and metastatic adenocarcinomas of the lung [15]. TTF-1 is a tissue-specific homeodomain-containing transcription factor that plays an important role in the early embryonic differentiation and morphogenesis of both lung and thyroid. It regulates the expression of surfactant apoproteins (A, B, C) and Clara cell antigens. In adults, it is almost exclusively expressed in thyroid and pulmonary epithelial cells. TTF-1 expression can be easily determined by IHC and it is highly specific in differentiating lung epithelial cancer (TTF-1 positive) from lung metastases [16]. It should be underlined that TTF-1 is reported to be positive in about 75% of adenocarcinoma (ADC) but not in squamous cell carcinoma (SCC). Moreover, regarding ADCs, TTF-1 expression is associated with solid and invasive mucinous subtypes and a lower frequency of epidermal growth factor receptor (*EGFR)* mutations. Overall, it defines a subgroup of lung ADC characterized by unfavorable prognosis. Napsin A is an aspartate protease and upregulated by TTF-1 in type II pneumocytes. Its expression has been shown to be useful in combination with TTF-1 in differentiating primary lung carcinoma from pulmonary metastases of extra-thoracic origin [17]. It should be underlined that some ADC markers may also be expressed in a small minority of lung metastases of distant primary epithelial tumors. For instance, TTF-1 expression has been documented in breast [18], ovarian [19] and hepatocellular [20] cancers. Similarly, increased *EGFR* expression occurs in a fraction of patients who have renal cell carcinoma (RCC) with an unfavorable histologic phenotype [21]. Overall, this behavior may be problematic when those staining tools are used in differential diagnosis between lung cancer and lung synchronous/metachronous metastases from different site of primary origin. 

Neuroendocrine differentiation of lung tumors is orchestrated by complex pathways. Among them, INSM1 (Insulinoma-associated protein 1) is a zinc-finger transcriptional factor originally isolated from pancreatic insulinomas [25]. It is inactivated by HES1 (Hairy and Enhanced of Split-1) transcription factor and promotes the expression of three neuroendocrine molecules: chromogranine A (CHGA), synaptophysin (SYP), and neural cell adhesion molecule 1 (NCAM1) via activation of transcription factors, such as Achaete-Scute family basic helix-loop-helix (bHLH) transcription factor 1 (ASCL1) and neural transcription factor BRN2. INSM1 is emerging as a novel, sensitive and specific IHC marker that may serve as a first-line marker of neuroendocrine differentiation. Moreover, IHC is applied to detect protein expression levels resulting from several genetic alterations. In the case of primary lung cancer, they mainly affect tyrosine kinase rearrangements and immune checkpoints. Anaplastic lymphoma kinase (A*LK*)-rearranged lung cancers are most often TTF-1 positive tumors, characterized by an acinar pattern with mucin/signet-ring cell morphology. These tumors define the 4–5% of ADC occurring in non-/light-smoker and young patients [26]. Immunohistochemistry is also a cost-efficient approach to detect *ROS-*rearrangements in non-small-cell lung cancer (NSCLC), although confirmation through fluorescent in situ hybridization (FISH) is required in positive samples. The lungs can also be site of growth of metastatic lesions of unknown primary origin. In this case, the conventional approach imposes to increase the diagnostic potential of finding a putative tissue of origin, by defining—in addition to immunostaining—the molecular profile of the lesion [27] with the underlying rationale of treating the lesions as a high-grade metastatic tumor of the predicted site of origin [28]. In this setting, the immunophenotype could be helpful in suggesting a probable lineage of origin of the metastatic mass [29]. Strictly speaking, CUPs are properly defined as carcinomas, the vast majority of which is represented by adenocarcinomas (90%), with squamous cell carcinomas and undifferentiated carcinomas being considerably less frequent. The inclusion of sarcomas, lymphomas and melanomas of unknown primary is sometimes reported, although in those cases the lineage of origin is clearly solved [30,31].

## 3. The Pulmonary Metastatic Niche and Microenvironment 

**Figure 3:**
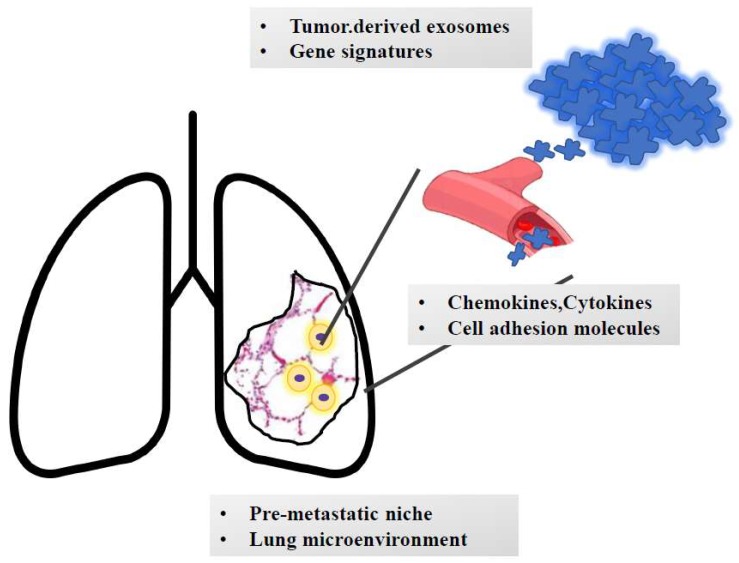
The journey of metastatic clones towards the lungs. The growth of metastatic masses into the lung parenchyma is orchestrated by tumor-derived signals (e.g., exosomes and genetic signatures). Once detached from the primary mass, metastatic clones invade blood (or lymphatic) vessels. Based on the interaction with adhesion molecules and based on the cross talk with chemokines and cytokines, cells reach the lung parenchyma and extravasate and colonize the pre-metastatic niche. Here, they undergo an epithelial-to-mesenchymal transition and interact with the surrounding stroma, which contributes to cell survival and growth. Moreover, smoke-induced chronic inflammation and hypoxia promote macrophage recruitment and immunotolerance.

The process of metastatic dissemination may already begin before cells migrate from a primary tumor mass and involves distinct steps. Genetic and phenotypic instability are among the earliest characteristics of transformed cells. Cancer cells are more prone to mutate and undergo phenotypic drift than their normal counterparts. Thus, genetic instability, coupled with a Darwinian type of selection—in which only the fittest survive—results in populations resistant to normal homoeostatic growth controls, immune attack, and environmental restraints. The rate of progression varies, and, within any neoplastic mass, subpopulations with different malignant potential can be identified. Local smoke-induced chronic inflammation may contribute to increased metastatic growth as has been demonstrated for lung metastases from breast cancer both in mice and patients [32,33]. The pro-metastatic effect played by smoking exposure is related to the activation of the ubiquitin- chemokine receptor type 4 (CXCR4) () pathway [34], high tissue levels of E-selectin [35], activation of the nuclear factor kappa-light-chain-enhancer of activated B cells (NFκB) signaling in pneumocytes [36], increased chemokine ligand 2 (CCL2) () expression and macrophage infiltration in the lung microenvironment [37]. Moreover, lung alveolar cells induce chemokine secretion, which recruit neutrophils. The latter, through the synthesis of arachidonate 5-lypoxigenase (ALOX5)-dependent leukotriene, may promote survival and proliferation of leukotriene B4-expressing metastatic clones [38]. Neutrophils can also secrete cathepsin G and elastases which further facilitate metastatic growth [39]. Overall, these signaling cascades allow the establishment of immunotolerant niches which promote the growth of neoplastic masses. Indeed, not all tumors are metastatic, nor are all cells within so-called metastatic tumors capable of metastatization. Recent evidence suggests that tumor cells might begin conditioning distant tissues for colonization by establishing a so-called “pre-metastatic” niche [40]. The complex biological program leading to lung metastatization is summarized in Figure 3. Since the new microenvironment of the metastatic site differs from that of the primary mass, the cells can die or survive; in the latter case, if proliferation is balanced by apoptosis, they remain clinically undetectable (dormant micrometastases); more often they display a relentless growth activity. Disseminated cells which have reached the lungs can undergo dormancy, mainly because they do not interact with the new parenchyma [41,42]. This behavior can occur even after a long period of latency after removal of the primary tumor. Different cancer types exhibit a wide variability in the length of latency: typically, short for lung cancer (only few weeks), while long for prostate and breast estrogen receptor (ER)-positive cancers (years/decades). Growing efforts are now directed towards targeting dormancy as a novel potential therapeutic approach to reduce the risk of cancer recurrence and dissemination [43]. Moreover, lung specific factors, stroma cells, extracellular matrix (ECM), cytokines and growth factors may affect metastatic cell survival and proliferation. To overcome the obstacles associated with the novel microenvironment, metastatic cells use cell-autonomous traits that facilitate homing and survival by altering gene of Rous sarcoma virus (SRC) tyrosine kinase signaling or the p38 and extracellular signal-regulated kinase-1 (ERK) mitogen-activated protein kinase (MAP) kinase signaling pathways and acquire a stem-cell like genetic profile, regulating the pre-metastatic lung niche. Tenascin C is another example of an ECM protein secreted by breast cancer metastatic cells to create a supportive niche in the lungs [44]. Various stroma cells, including fibroblasts, neutrophils and vascular endothelial growth factor receptor 1 (VEGFR1)-positive bone marrow-derived hematopoietic progenitor cells, also play a central role in niche preparation. In a spontaneous prostate cancer metastasis model, mitogen-activated protein kinases 7 (MKK7) suppress formation of lung metastases by inhibiting the ability of disseminated cells to colonize the distant tissue [45]. Factors secreted by the microenvironment, such bone morphogenetic proteins (BMPs) and growth arrest-specific 6 (GAS6) produced by osteoblasts, can directly inhibit disseminated tumor cell (DTC) proliferation. Single 4TO7 cells enter arrest immediately upon infiltrating the lung and are therefore unable to form micrometastatic lesions [46]. Currently, cellular dormancy is mainly associated with solitary cells, while dormant macrometastatic lesions are considered to consist of actively proliferating cells balanced by the same number of apoptotic cells. Stephen Paget’s hypothesis formulated in 1889 according to which metastases development depends on crosstalk between selected cancer cells (the “seed”) and the specific microenvironment (the “soil”) still holds forth today. The metastatic potential arises from multiple biological processes that eventually instruct cells to detach (EMT), migrate and evade cell death. During distant dissemination, cells acquire metastatic competence defined by a mesenchymal gene expression pattern. This allows cells to detach from the primary site of growth, invade blood vessels, cross their walls and colonize a distant organ/tissue [47]. Morphogenesis and metastasis seem to arise from the same genetic program that instructs cells to undergo a form of programmed cell death known as *anoikis* when they detach from the surrounding extracellular matrix [48]. Metastatic cells more frequently migrate collectively to form a structural and functional unit based on a strict cell-cell interaction and active crosstalk [49]. Moreover, it has been documented that some cancer types preferentially metastasize to specific organs. The determinants of such a biological program include: (i) cell-intrinsic determinants, e.g., chemokines, cytokines; (ii) adhesion and extracellular matrix molecules, e.g., tenascin and periostin; (iii) tumor-derived exosomes [50]. The latter are small membrane-bound vesicles of endocytic origin that can transport molecules including proteins, DNA, RNA and non-coding RNA from one cell to another, thus enhancing the horizontal exchange of genetic information [51]. In the cancer setting, tumor-derived exosomes have been demonstrated to be taken up by organ-specific cells to prepare the pre-metastatic niche, implying that the analysis of the expression of exosomal integrins could be used to predict organ-specific metastases [52]. In detail, lung-tropic exosomes expressing the integrins (ITG) such as ITGα6β4 and ITGα6β1 preferentially interact with S100A4-positive fibroblasts and surfactant protein-positive pneumocytes [14]. Moreover, it has been experimentally demonstrated that small nuclear RNAs enriched in tumor exosomes can activate toll-like receptor 3 in alveolar type II cells and consequently induce chemokine secretion and neutrophil recruitment in the lung [53]. These steps are critical for initiating the formation of a metastatic niche in the lung and demonstrated organ-site-specific tropisms of metastatic cells. In order to develop the metastatic mass, circulating neoplastic cells need to adhere to endothelial walls and extravasate to reach the lung parenchyma. Activation of VCAM1 has been reported in lung secondary masses from breast cancer. It is expressed in endothelial cells and can initiate trans-endothelial migration by binding certain integrins such as α4β1 and α4β7. The latter induce the activation of GTP-ase Ras-related c3 botulinum toxin substrate 1 (Ras-related C3 botulinum toxin substrate 1, RAC1) which subsequently induces modification of the cytoskeleton network and facilitates cell migration [54]. The scaffold protein src-suppressed C-kinase substrate/gravin/A-Kinase Anchoring Protein 12 (SSeCKS/Gravin/AKAP12) is known to control metastasis-associated protein kinase C (PKC) and SRC signaling through direct scaffolding activity. The SSeCKS complex is deregulated in lung metastases from melanoma [55]. Colony stimulating factor 1 (CSF1) is known to act as mediator of lung metastases: those tumors developing in CSF1-expressing mice have been shown to recruit macrophages and to feature a highly invasive potential which enhances the formation of lung neoplastic colonies. This effect is mainly mediated by epidermal growth factor (EGF) released by macrophages which induce CSF1 secretion by tumor cells and recruit macrophages by interaction with colony stimulating factor 1 receptor (CSFR1) [56,57]. It has also been experimentally demonstrated that breast cancer cells that overexpress chemokine (C-X-C motif) ligand 1 (CXCL1) and 2 by transcriptional hyperactivation or 4q21 amplification display increased survival, the onset of lung metastases and enhanced chemoresistance through myeloid cell recruitment [41]. The pulmonary extracellular matrix (ECM) determines the tissue architecture of the lung and provides mechanical stability and elastic recoil, which are essential for physiological lung function. Successful metastasis formation requires early remodeling of the lung ECM in the metastatic niche. The correlation between expression of the extracellular matrix glycoprotein tenascin C and breast cancer metastatization to the lung has been reported, with tumor-related tenascin C being essential in early phases of metastatic onset [58]. Later, the tumor stroma becomes the source for tenascin C which maintains the metastatic growth. The extracellular matrix protein periostin is also involved in lung metastatic development since periostin knock-out mice develop mammary tumors with a reduced number of lung metastases compared to periostin wild-type animals [59]. Indeed, periostin and tenascin C enhance the Wingless-related integration site (WNT) and NOTCH signaling. The latter pathway is significant in maintaining stem/progenitor properties and in enhancing the viability of cancer cells [60]. Tenascin C also increases the concentration of growth factors such as EGF, vascular endothelial growth factor (VEGF), fibroblast growth factors (FGF) which capable of promoting growth of metastatic masses [61]. Prior reports have documented the presence of metastasis-suppressive niches. Metastatic cells might express the glycoprotein prosaposin, which, in turn, can reprogram myeloid cells in the lungs to express thrombospondin-1 (TSP1), thereby generating suppressive niches in animals [62]. These results unveiled the plasticity of myeloid cells, which, based on the context, promote or inhibit metastases, and suggest that prosaposin could be a potential therapeutic agent against metastatic cancer. 

A critical issue in cancer progression is related to intratumoral heterogeneity and hierarchical organization. The tiny fraction of cancer stem cells (CSCs) have been reported to be the ones involved in carcinogenesis, metastasis, recurrence, and treatment resistance [63]. The interaction between organs that are targets for metastases and CSCs has been reported in a breast cancer study. The report showed that the bone morphogenic protein inhibitor COCO regulates the cycle of tumor dormancy and activity in the lungs and promotes metastasis of breast CSCs to the lungs but not to the bone or brain [64]. Although the vast majority of circulating tumor cells may possess intrinsic defects that preclude them from surviving or undergoing active proliferation in the lung, the ones that are fated to give rise to clinical metastases, the metastasis-initiating cells, feature a stem-cell like phenotype and face strong antimetastatic signals originating from the parenchyma of this organ [65]. Moreover, the specific organ microenvironment influences CSC growth rate, as reported in *in vivo* models of lung and liver metastases from pancreatic cancer [66]. It has been also demonstrated that subsets of CSC express distinct markers confirming that different subpopulations have the potential to metastatize efficiently in different target organs. Indeed, migrating CSC isolated from colorectal cancer patients had been classified based on expression of two distinct surface markers CD110 and CDCP1 which specifically promote adhesion to lung epithelium and liver cells respectively, giving rise to a highly organ-specific pattern of dissemination [67].

## 4. Genetic Signature Associated to Metastatic Tropism to the Lungs

The process of metastatic dissemination begins when malignant cells leave the primary mass and start to move. It is now accepted that neoplastic progression is associated with a combination of genetic and epigenetic events. Cancer is a genetic disease and this pathogenic concept is the basis for a new classification of tumors, based on the presence of definite genetic lesions to which the clones are addicted [68]. The metastasis progression gene signatures emerge very early in the tumor life. The possibility to analyze gene expression profiles in primary tumors and to compare different signatures in various cancer types, has demonstrated that metastases rely—at least in part—on gene mutation and gene regulation events that occur in the vast majority of cells which constitute the primary tumor mass [69], with expression profiles from primary tumors being similar to those derived from metastatic samples [70]. It has also been demonstrated that the signature derived from lung metastases of breast cancer is not only related to the clinical outcome, but quite unexpectedly, to the tumor size [71]. Thus, if the metastatic signature is associated with primary tumor growth, the metastatic phenotype will not really derive from a selective advantage, but rather from the footprint on gene expression left from metastatic population. Gene expression signature analysis describes multiple tissue-specific metastatic programs. Interestingly Golub and colleagues [67] analyzed twelve metastatic adenocarcinoma nodules of different origin (lung, breast, prostate, colorectal, uterus, ovary) and compared them with the expression profiles of 64 primary ADCs, representing the same spectrum of tumor types obtained from different individuals. This comparison allowed the identification of an expression pattern of 128 genes that best distinguished primary and metastatic adenocarcinomas [67]. Moreover, a prognostic 28-gene signature has been identified by the analysis of microarray expression data in primary cutaneous melanoma samples and has been validated as an independent predictor of metastatic risk [72]. Similarly, the gene expression profile analysis of a subset of primary breast tumors generated a unique 14-gene signature (*WDR6, CDYL, ATP6V0A4, CHAD, IDUA, MYL5, PREP, RTN4IP1, BTG2, TPRG1, ABHD14A, KIF18A, S100PBP and BEND3*) able to predict the risk of development of visceral organ metastases [73]. Interestingly, functional characterization of lung metastases gene signatures reveals that they contain little information on growth factors and their receptors, that are known to orchestrate metastatic growth [74]. Most of the products of genes that are differentially expressed during tumor progression are defined as defense genes and extracellular matrix. This finding confirms that metastatic growth derives from a complex tumor–host interaction. Notably, none of the factors alone are sufficient to determine if the secondary mass will develop or not. 

The evolution process is regulated by the following features: types of genetic aberrations present; mutation rates; extent and intensity of selection; and finally, the heterogeneity of tumor cells subclones [75]. Thus, the metastatic process, instead of being a unidirectional phenomenon, emerges as a multidirectional process in which tumor cells can seed to distant sites and come back to the primary site. The latter, namely the self-seeding process, is critical for both tumor and metastatic growth [76]. Limited data are available on specific neoplastic seeding in the lung parenchyma. It has been shown that hypoxia in the primary tumor (breast cancer) associated with high levels of HIF1α upregulates the expression of the lysil-oxidase gene; the encoded protein binds to collagen in the lungs, thus promoting of myeloid cells to develop tolerant and pro-metastatic niches [77]. It has been demonstrated that clusters of circulating tumor cells (CTCs) are oligoclonal precursors of breast cancer metastases. Authors tagged breast and melanoma cell lines with fluorescent proteins and/or luciferase. The MDA-MB-231 human breast carcinoma cell line (MDA231) and its variant, selected via intravenous inoculations for enhanced lung colony formation (MDA231-LM2) [56], were primarily used in reseeding or cross-seeding experiments. Tagged and untagged tumor cells were injected separately into orthotopic contralateral sites. MDA231-LM2 cells were highly efficient in disseminating and self-seeding a contralateral MDA231-LM2, but they were not reported to spontaneously seed to lungs from the primary tumor. Authors concluded that spontaneous seeding of a tumor mass is less restrictive than seeding of target organs. The interleukin 6 (IL-6) and interleukin 6 (IL-8) cytokines produced by contralateral human tumors served as CTC attractants and this may explain the preferred targeting of spontaneous tumors compared with the lungs. Alternatively, enhanced lung colonization selected through forced intravenous inoculation [61] may exacerbate cell traits that do not entirely recapitulate organ-specific metastases. Due to the rapid development of new and high-throughput technologies, novel metastasis candidate genes will be identified with both clinical and therapeutic implication. More recent gene expression analysis has allowed the identification of genetic signatures associated with lung metastasization. Inhibitor of cell differentiation 1 (*ID1)*, matrix metallo-proteinase 1 (*MMP1)*, chemokine CXC motif ligand 1 (*CXCL1*), prostaglandin-endoperoxide synthase (*PTGS2*), vascular cell adhesion molecule-1 (*VCAM1*), and epiregulin (*EREG*) were among the genes that promote lung metastasis in animal models carrying breast carcinoma, and their differential expression was able to differentiate among breast cancer patients, those with lung metastases vs those without. Notably, gene expression of ID1 promotes formation of lung metastases by itself in animal models and is highly expressed in samples from breast cancer patients with lung metastases. Indeed, the DNA-binding protein inhibitor ID-1 is a protein that is encoded by the ID1 gene and its activation promotes breast cancer dissemination by modulating S100A9 expression [78]. The latter is a calcium-binding protein with multiple ligands and post-translational modifications that is involved in inflammatory events and the initial development of metastatic disease [79]. With respect to the analysis of human cancer samples, it has been shown that the comparison of lung and non-lung metastases from breast cancer identified 21 differentially expressed genes [80] which mainly encode adhesion molecules, resulting in cell to cell interactions and thus facilitating lung colonization. Among them are integrins (*ITGB8*), cadherins (*CDH3*), desmosomal proteins (*DSC2*), and focal adhesion molecules (*FERMT1*). 

Although a detailed description of the role of micro-RNAs (miRNAs) in the metastatic cascade goes beyond the scope of this review, it should be noted that several *in vitro* and *in vivo* studies have described metastases-associated miRNAs signatures. MiRNAs might act in each phase of distant tumor spread by regulating cell invasion and migration capacity as well as growth of distant masses [81]. With respect to the epithelial-to-mesenchymal transition (EMT), a step required for metastatic dissemination, the miR-200 family has been reported as a key mediator in regulating the expression of E-cadherin [82]. In detail, miR-200 family act by inhibiting the EMT and by maintaining the epithelial phenotype through direct targeting of transcriptional repressors of *E-cadherin, zinc finger E-box binding homeobox 1 (ZEB1)* and *zinc finger E-box binding homeobox 2 (ZEB2) genes.* Moreover, many miRNAs control the angiogenic process. Among them, miRNA-29c overexpression is known to inhibit angiogenesis by downregulating VEGF [83]; miRNA-519c is known to attenuate angiogenic activity of endothelial cells and to suppress angiogenesis and metastasis formation by reducing HIF-1α levels [84]. Interestingly, recent data suggest that miRNA signatures might display tissue/organ specificity. Osaki et al. reported that miR-143-3p expression decreased in a metastatic osteosarcoma cell line (143B) and primary osteosarcoma tissues with lung metastasis [85]. By comparing samples from patients without metastases and sample from those with lung metastases, Sasaki et al. showed that increased expression of miR-27a and decreased miR-95-3p and miR-195 expression and miR-133 dysfunction are associated with which cancers develop lung metastasis [86]. In colorectal cancer, the overexpression of miR-885-5p resulted in significantly induced cell migration, invasion, formation of stress fiber *in vitro* and was associated with the development of liver and lung metastases in *in vivo* models [87]. Finally, miRNA signatures have also been associated with the pattern of lung metastatization, able to distinguish cancer progression with oligometastes vs. polymetastatic evolution by modulating the axon guidance, cancer metastasis, and proteoglycan pathways [88].

## 5. Mechanical Interactions between Tumor Masses and Surrounding Microenvironment

Traditionally, cancer research has primarily focused on the biological characterization of the processes involved in metastasization. In recent years however, concomitant marked alterations in the mechanical phenotype of the cancer cells and the surrounding microenvironment have become increasingly recognized as pivotal steps [89,90,91,92,93]]. In what Kumar et al. have dubbed a “force journey” [94], there appears to be a dynamic mechanical reciprocity between the tumor cell and its microenvironment governing the cell’s progression through the various stages, comprising detachment from its neighboring cells, invasion of the surrounding parenchyma, intravasation into the vascular system, survival while in circulation, extravasation and growth of a secondary mass at the distant target site. Like metastases of known primary tumors, metastases in the context of CUP are expected to continue, if not escalate, this mechanical interplay with their microenvironment, in turn enabling metastatic cells to re-enter the circulation and seed new metastases or re-seed back to existing metastases [95,96]. While a detailed discussion of the full repertoire of mechanical interactions during the different phases of metastasization is beyond the scope of this review, we briefly discuss potential ways of interaction between pulmonary metastatic masses and the surrounding extracellular matrix (ECM). On a macroscopic level, local expansion of a tumor mass exerts compressive forces on the ECM, thereby constricting flow in the vasculature, lymphatic system and interstitial space. The elastic Young’s Modulus, a measure of deformability of a material in response to mechanical stress, can be used to quantify the compliance of a tissue [97,98]. Lung tissue has been reported to have an approximate bulk Young’s Modulus of 5–6 kPa, finding itself towards the lower end of the spectrum ranging from highly-compliant brain tissue (0.3–0.5 kPa) to poorly-compliant skeletal muscle (50 kPa) [99]. Hence, in the setting of compliant pulmonary tissue, the outward projecting compression force due to expansion of the tumor mass is thought to facilitate cell detachment and subsequent invasion into the parenchyma [100]. Voutouri et al. estimated that tumors should be at least 1.5 times stiffer than their surrounding normal tissue in order to exert a sufficient compressive force to overcome confinement by the host tissue [101]. Such compression forces progressively shrink the surrounding interstitial space, thereby concentrating tumor-promoting growth factors and cytokines shed into that very space [102]. In addition, these stresses may play a role in tumor angiogenesis, either through direct upregulation of VEGF secretion or indirectly as a result of induced tissue hypoxia [103,104,105]. The aforementioned compressive forces, and hence their effects, may be further exacerbated by tumor-induced progressive stiffening of the adjacent ECM. Despite vast secretion of metalloproteinases and matrix digestion, the ECM in the immediate vicinity of a tumor is typically rather dense due to increased matrix deposition, collagen crosslinking through enzymes such as lysyl oxidase [106,107,108], and an intense fibrotic response known as desmoplasia [109]. Pathological fibrosis, whether it be cancer or otherwise, promotes cell invasion and migration by elevated tissue stiffness [110,111,112,113]. This stiffening is routinely exploited clinically to detect tumors through physical palpation and by commonly used imaging techniques, which derive their contrast from mechanical compliance differences within tissue [114]. While a thorough characterization of ECM stiffness has not been reported for the lung, a 5- to 20-fold increased stiffness compared with normal mammary gland has been quantified for breast carcinomas [56]. Furthermore, occurrence of ECM stiffening around metastatic lesions at similar levels to that around the primary tumor has been shown for pancreatic cancer [115]. ECM stiffening activates mechano-transduction signaling pathways, which drive force-dependent integrin clustering [116] and subsequent increased focal adhesion assembly and ROCK-generated disruption of adherens junctions [117,118] through enhanced ERK- and Rho-dependent cytoskeletal contractility [119,120,121,122]. Novel drugs targeting these pathways have sparked renewed enthusiasm for future treatments aimed to tilt the balance in the battle against metastasization [123].

## 6. Concluding Remarks

Secondary neoplastic lesions commonly arise in the lung parenchyma: integration of histology, immunohistochemistry and imaging can help in identifying the primary site of origin, on the one hand, or in defining them as CUPs by *ad excludendum* diagnosis, on the other hand. The complex network which regulates pulmonary metastatic growth is emerging as potential diagnostic and therapeutic target. Particularly, this landscape is characterized by both a tumor-reprogrammed microenvironment and the activation of immunosuppressive mechanisms. Moreover, the unique biophysical features of the lung play a role in the expansion of secondary masses. Recent advances and emerging concepts, coming from experience gathered from immunotherapy, have shed new light on the therapeutic potential of targeting the so-called lung pre-metastatic niche. Overall, the clinical implications of these approaches are represented by a better stratification of breast cancer patients and early identification of those women at higher risk of developing secondary lung lesions. Further investigations are required to address relevant questions, namely if all lung metastatic types behave in the same way and which genetic elements define the lung tropism in circulating tumor cells.
